# The direct oxidative diene cyclization and related reactions in natural product synthesis

**DOI:** 10.3762/bjoc.12.200

**Published:** 2016-09-30

**Authors:** Juliane Adrian, Leona J Gross, Christian B W Stark

**Affiliations:** 1 Fachbereich Chemie, Institut für Organische Chemie, Universität Hamburg, Martin-Luther-King-Platz 6, 20146 Hamburg, Germany

**Keywords:** asymmetric synthesis, natural products, oxidation catalysis, tetrahydrofurans, total synthesis

## Abstract

The direct oxidative cyclization of 1,5-dienes is a valuable synthetic method for the (dia)stereoselective preparation of substituted tetrahydrofurans. Closely related reactions start from 5,6-dihydroxy or 5-hydroxyalkenes to generate similar products in a mechanistically analogous manner. After a brief overview on the history of this group of transformations and a survey on mechanistic and stereochemical aspects, this review article provides a summary on applications in natural product synthesis. Moreover, current limitations and future directions in this area of chemistry are discussed.

## Introduction

### Scope of this article

After a concise introduction on the history and mechanistic aspects of the title reaction, the primary aim of the present review article is to summarize all relevant applications in natural product synthesis. The main text of this article is ordered by compound classes, so that tactics can easily be analysed and compared and similar applications can be condensed (both in the text and in the corresponding schemes). Methodology driven investigations as well as mechanistic studies are not the main focus of this review but may be mentioned in the introductory section. Likewise, syntheses of fragments of natural products applying an oxidative cyclization protocol [1–2] and sequential epoxidation/cyclization procedures [3] are not in the scope of this article and are therefore not covered. Previous review articles concerning oxidative diene cyclization chemistry can be considered in complement [4–6].

#### Oxidative cyclization – Historical background

**Scheme 1:**
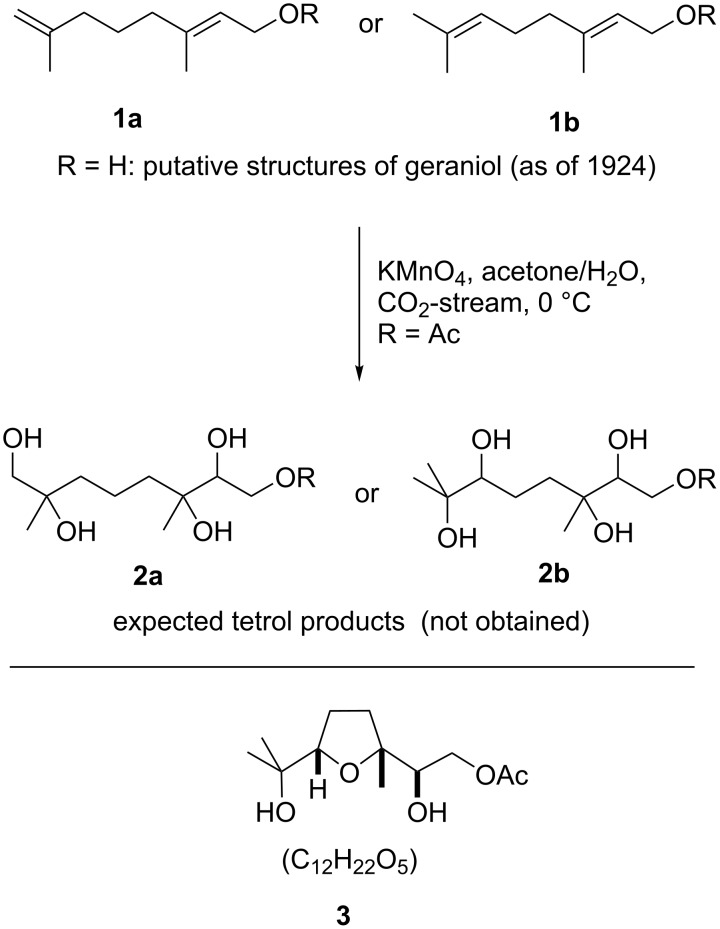
Putative structures of geraniol **1a** (R = H) or **1b** (R = H) (in 1924), their expected dihydroxylation products **2a** or **2b** and the true structure **3** as determined by Klein and Rojahn in 1965 [8].

In 1924 Kötz and Steche reported on an investigation of the constitution of the monoterpene geraniol (**1**, R = H) [7]. Though the overall structure was known at that time, the position of one of the two C–C-double bonds within that natural product was in dispute (Scheme 1). Thus, the authors subjected a derivative (geranyl acetate (**1**, R = Ac), Scheme 1) to an aqueous solution of permanganate to dihydroxylate both double bonds in order to elucidate the structure. Elemental analysis of the crystalline reaction product (“*Der reine Stoff bildet prächtige Krystalle …*“ [7]) revealed that not one of the expected tetrols **2a** or **2b** (Scheme 1) but rather a cyclic anhydro compound seemed to be the result. Though a set of further reactions were carried out on this oxidation product, it proved not possible to establish its structure. It was not until 1965 when Klein and Rojahn at the flavours and fragrance company DRAGOCO (now Symrise AG) in Holzminden, northern Germany, reinvestigated the conversion of geranyl acetate (**1b**, R = Ac) with permanganate and were able to determine that the actual product is a 2,5-bis(hydroxymethyl) THF (**3** in Scheme 1, the general structure of which is today often as a simplification referred to as “THF diol”) [8]. In addition, they found that this reaction proceeds with high stereoselectivity (vide infra) and demonstrated that the reaction is not only limited to terpenes such as geranyl- (**1b**, R = Ac) or neryl acetate but seemed to be fairly general to other 1,5-diene substrates. Finally, they speculated on possible intermediates which may account for the outcome and the overall stereoselectivity of this unusual reaction. A mechanism, however, was not provided (vide infra).

To date it is firmly established that in addition to the permanganate-mediated reaction, both ruthenium- as well as osmium tetroxide mediate the same transformation (cf. Scheme 3) and that these reactions can, contrary to the original permanganate-promoted process, be run in a catalytic fashion. All published protocols using the three different d^0^-metals are highly diastereoselective (vide infra) and have been shown over the past decades to be applicable to a broad range of starting materials.

#### Mechanistic aspects, stereochemistry and substrate scope of the direct oxidative diene cyclization

**Scheme 2:**
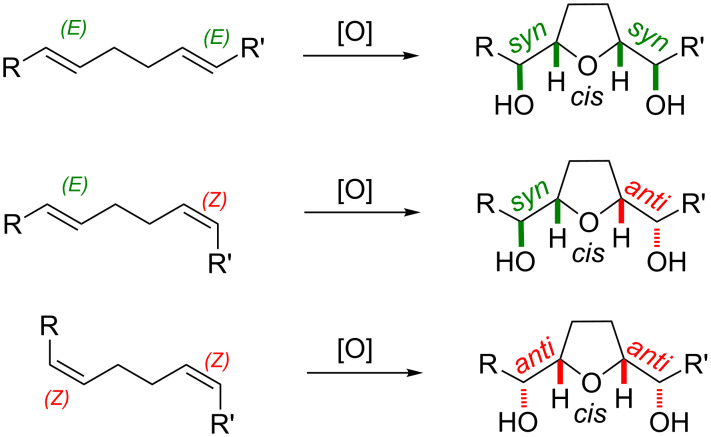
Correlation between the substrate double bond geometry and relative stereochemistry of the corresponding THF diol products.

**Scheme 3:**
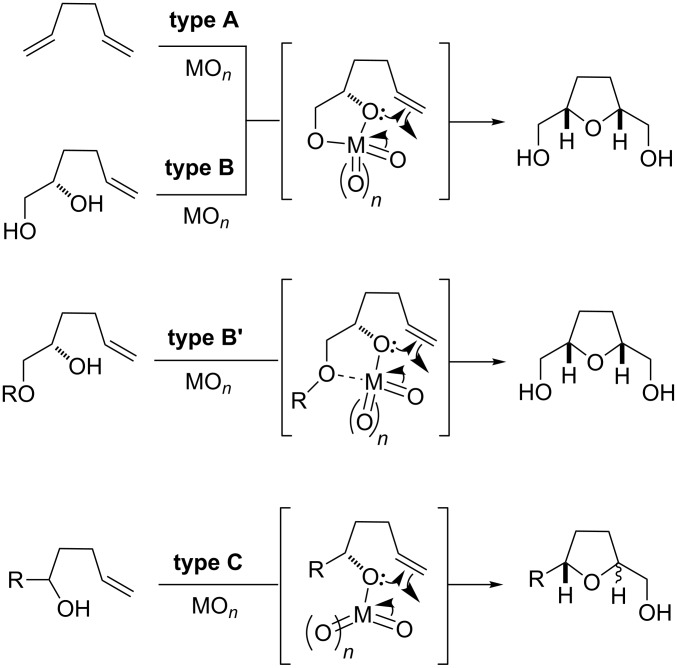
Mechanisms and classification for the metal-mediated oxidative cyclizations to form 2,5-disubstituted THFs.

Intrigued by the unique chemistry reported by Klein and Rojahn [8], several research groups initiated programs in order to shed light on the stereochemical course and mechanism of what appeared to be a direct oxidative diene cyclization. After a controversial debate from the early years of the discovery until the 1980s, it was finally broadly accepted that the overall reaction is a result of two consecutive *syn*-stereospecific [3 + 2]-oxidative cycloadditions (cf. type A mechanism; Scheme 3) [9–11]. Therefore, the double bond geometry of each of the two reacting double bonds translates directly to the relative stereochemistry of the vicinal hydroxy ether motif of the product (Scheme 2). The stereochemistry across the THF ring is set in the cyclization event. As a result of geometrical constraints it is usually predominantly or even exclusively *cis* (Scheme 2) – a fact that has recently been corroborated through density functional theory calculations both by Strassner and co-workers (Mn(VII) and Os(VIII)) [12–13] and by Kirchner and co-workers (Ru(VIII)) [14]. Reasonable fractions of the *trans*-THF isomer can be produced using ruthenium tetroxide in specifically optimized solvent compositions [15] (for other means to obtain the *trans*-isomer from *cis*-THFs see the examples section below).

For all three procedures (using Mn(VII), Ru(VIII) and Os(VIII)) the scope of the reaction is very broad and a large number of 1,5-dienes with any kind of substitution pattern and double bond geometry have been used as substrates [4–6,16–17]. In addition, for Ru(VIII) [17–19] and Mn(VII) [21] it has been shown that also 1,6-dienes serve as substrates and can thus be directly converted to tetrahydropyrans [20–22]; ruthenium tetroxide even oxidizes 1,7-dienes to oxepans [23]. However, it has to be noted that the latter transformations do not have the same broad substrate spectrum as has been demonstrated for 1,5-diene precursors and there are no applications to natural product synthesis thus far.

A particularly fruitful extension of the direct 1,5-diene oxidation methodology (and due to mechanistic similarities also within the scope of this review) is the oxidative cyclization of 5,6-dihydroxyalkenes. This reaction has been reported to be catalyzed by Os(VI), Ru(VII) and Cr(VI) [24–26] and can be termed type B oxidative cyclization (as opposed to the direct oxidative cyclization of 1,5-dienes, referred to as type A reaction; cf. Scheme 3). In this case the diol and the metal oxide form a glycol ester intermediate which then undergoes an intramolecular oxidative addition to a remote double bond. Thereby, type B oxidative cyclizations converge to the same (or very similar) reactive intermediate as is passed through in type A reactions (Scheme 3). A relevant advantage of this approach is that enantiomerically pure products can be obtained when enantiomerically pure diol starting materials are used. A subgroup of closely related starting materials may contain an alkyl ether instead of a free hydroxy group at C6 (R ≠ H in Scheme 3). The key intermediate and cyclization precursor may then involve a coordinative bond of that ether oxygen to the strongly Lewis acidic metal center. Due to the close relation to type B cyclizations, they can be classified as type B’ (Scheme 3). Reagents that mediate this type of reaction are Re(VII), Cr(VI) and Co(II) complexes [26–29]. Again, the observed efficiency and stereoselectivity for this class of oxidative cyclization reactions is high.

Another set of substrates are 5-hydroxyalkenes, starting materials, completely lacking the 6-OH-group (or ether oxygen donor). These compounds can only form mono-esters with the metal oxidant (Scheme 3). Therefore, they exhibit a different reactivity and a less ordered transition geometry in the oxygen transfer reaction and are thus categorized as a distinct class of oxidative cyclization, referred to as type C reaction. In fact, in these cases, mostly a *trans*-selectivity for the cyclization event is observed, due to the loose coordination. The most prominent oxidants to promote such type C reactions are Co(II) and Re(VII) complexes [27–29]. Reactions where a 5- and/or 6-(di)hydroxy group directs an oxidizing reagent to an internal alkene to form an epoxide followed by a subsequent cyclization are not covered in this article as these are different in mechanism since the oxidation and cyclization are two distinct events and do not occur in the same step [3].

The attraction to generate up to four chiral centers from a simple 1,5-diene precursor or up to two stereogenic centers when starting from 5,6- or 5-(di)hydroxyalkenes has progressively drawn the attention of synthetic organic chemists. These efforts have so far yielded some beautiful and persuasive results in the synthesis of natural products. The aim of the present review is to assemble key results of these applications and illustrate scope and limitations.

## Review

### Oxidative cyclizations in the synthesis of carbohydrates, amino acids and polyketide natural products

#### (+)-Anhydro-D-glucitol and (+)-D-chitaric acid

**Scheme 4:**
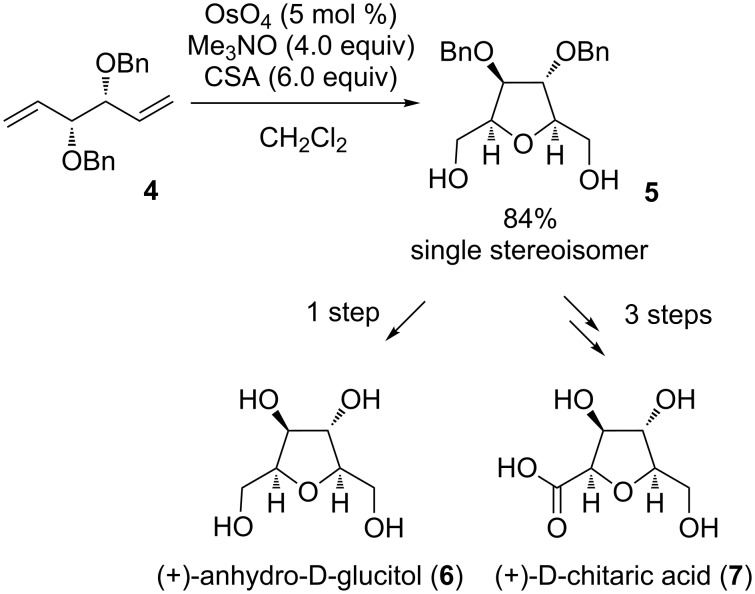
Synthesis of (+)-anhydro-D-glucitol and (+)-D-chitaric acid using an OsO_4_-mediated oxidative cyclization.

(+)-Anhydro-D-glucitol (**6**) was isolated from the mould fungus *Fusarium solani* as a phytotoxin against barnyardgrass and duckweed in 1996 [30]. The Donohoe group presented a total synthesis in 2003 using an Os(VIII)-catalyzed oxidative cyclization as the key step [31] (Scheme 4). Several other total syntheses of that natural product did already exist or followed [32–39].

Starting from the readily available *C*_2_-symmetric 1,5-diene **4** the 2,3,4,5-tetra-substituted THF diol **5** was obtained as a single stereoisomer with a yield of 84%, following the type A cyclization. Deprotection led to natural (+)-anhydro-D-glucitol (**6**) (Scheme 4). It was also possible to produce another carbohydrate using the same synthetic pathway. Thus, mono-protection of THF diol **5** followed by oxidation of the remaining free primary hydroxy group to the carboxylic acid and a final hydrogenolysis gave (+)-D-chitaric acid (**7**) with a yield of 30% over three steps (Scheme 4). Additional to the synthesis of Donohoe described above [31], two other total syntheses of (+)-D-chitaric acid have been reported [40–41].

#### Neodysiherbaine A

In 2001, the excitatory amino acid neodysiherbaine A (**14**) has been found in the marine sponge *Dysidea herbacea* by Sakai et al. together with the already known and closely related dysiherbaine [42]. Neodysiherbaine A (**14**) is a neurologically active compound that acts as a glutamate receptor agonist and shows epileptogenic properties. Contiguous to the isolation, the first synthesis has been carried out by the same research group [42] and several other syntheses followed [43–47].

**Scheme 5:**
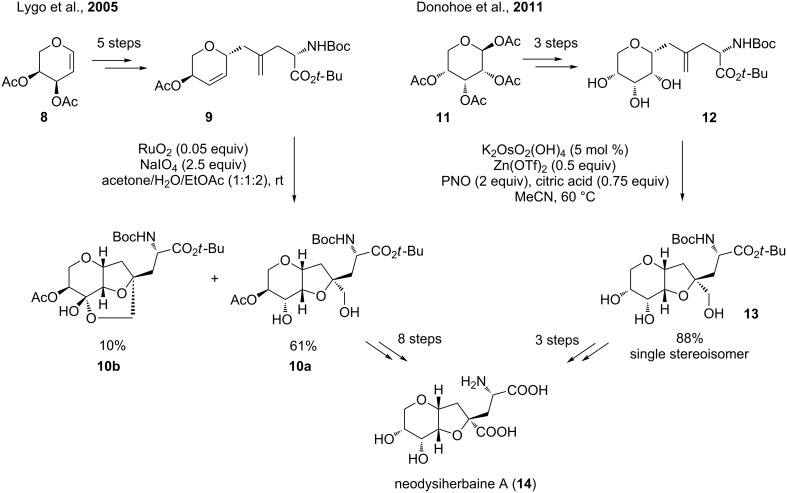
Total synthesis of neodysiherbaine A via a Ru(VIII)- and an Os(VI)-catalyzed oxidative cyclization, respectively.

The Lygo group chose an approach using a Ru(VIII)-catalyzed type A oxidative cyclization to form the THF motif of the natural product (Scheme 5, left) [48–49]. Starting from diacetyl-L-arabinal (**8**), 1,5-diene **9** was obtained, which was subsequently cyclized. The reaction yielded the desired THF diol **10a** in 61% as a single diastereoisomer together with over-oxidized **10b** as side product. The total synthesis was finally achieved from **10a** via some protecting group operations and an oxidation of the primary alcohol to the carboxylic acid [50–51].

In 2011, the Donohoe group developed a total synthesis of neodysiherbaine A (**14**) using an Os(VI)-catalyzed type B oxidative cyclization of a 5,6-dihydroxyalkene (Scheme 5, right) [52]. Commercially available β-D-ribopyranose tetraacetate (**11**) was converted to **12** via a Negishi coupling [53–54]. The oxidative cyclization diastereoselectively led to the THF diol **13** in 88% yield from which neodysiherbaine A (**14**) was obtained in a further three steps.

#### Ionomycin

**Scheme 6:**
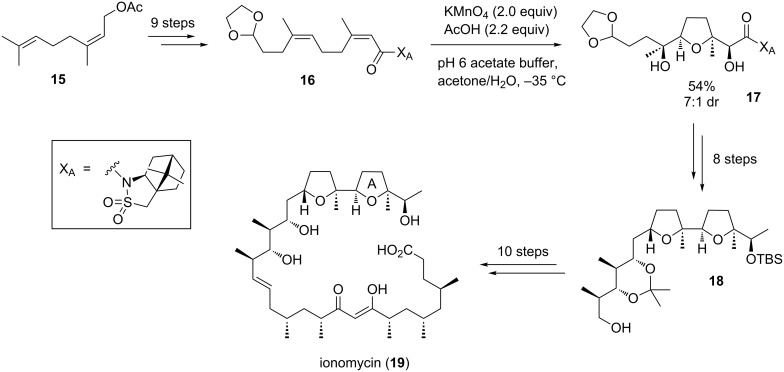
Formal synthesis of ionomycin by Kocienski and co-workers.

Ionomycin (**19**), an ionophore antibiotic isolated from *Streptomyces conglobatus* in 1978 [55–57], has a high affinity for divalent cations. It is commonly used to both modify intracellular Ca^2+^ concentrations and to investigate Ca^2+^ transport across biological membranes [58]. In 2011, Kocienski and co-workers reported on a formal synthesis of ionomycin using an auxiliary-directed, diastereoselective permanganate-mediated oxidative cyclization to introduce the THF ring A and four of its stereogenic centers in a single step (Scheme 6) [59]. A related approach had previously been featured as a key step in their synthesis of salinomycin, a commercially significant coccidiostat [2].

The required (*Z*,*Z*)-diene **16** was prepared from commercially available neryl acetate (**15**). The auxiliary-controlled, permanganate-promoted oxidation of diene **16** proceeded selectively at low temperatures, affording the corresponding diastereomeric THF diols as an inseparable mixture (dr 7:1, major stereoisomer shown in Scheme 6). Compound **17** could successfully be converted into alcohol **18**, an intermediate in the previously reported total synthesis of ionomycin (**19**) by Kocienski and co-workers [60] and also in the preceding syntheses developed by the group of Evans [61] and the Hanessian group [62], thus completing a formal synthesis of this polyketide. At this point it has to be mentioned that in 1987 the group of Weiler also used such a permanganate-promoted oxidative cyclization for the stereoselective synthesis of the THF unit in ionomycin [63]. Similarly, in 1980 Walba et al. reported on the B/C-ring fragment synthesis of monensin A, another well-known ionophore antibiotic, applying an oxidative cyclization approach using potassium permanganate [64].

#### Amphidinolide F

**Scheme 7:**
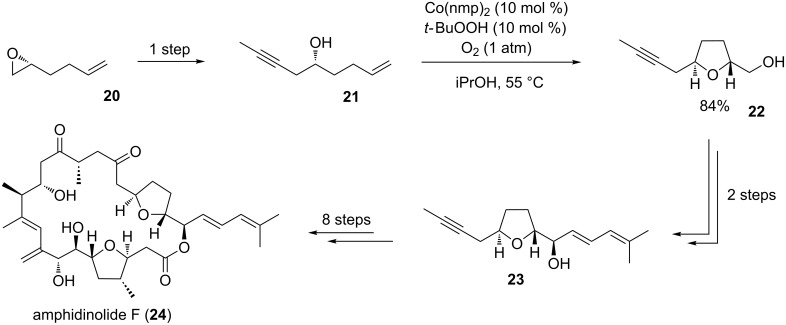
Total synthesis of amphidinolide F by Fürstner and co-workers.

Amphidinolide F (**24**) is a marine natural product isolated from the dinoflagellate *Amphidinium* sp. in 1991 [65]. The macrocyclic core of these highly cytotoxic secondary metabolites contains two 2,5-*trans*-substituted THF ring systems (Scheme 7) [66–67]. Despite significant efforts from various research groups, it took more than two decades from its isolation and characterization to the publication of its first total synthesis by Carter and co-workers in 2012 [67–68].

In 2013, the Fürstner group published a successful approach to amphidinolide F (**24**) applying an oxidative type C Mukaiyama cyclization reaction for the THF segment **22** (Scheme 7) [69–70]. Therefore, enantiomerically pure epoxide **20** was converted to 5-hydroxyalkene **21**, the oxidative cyclization precursor in this total synthesis. The subsequent cobalt-catalyzed cyclization reaction proceeded chemoselectively in the presence of the alkyne moiety and provided the *trans*-disubstituted THF **22** in high yield [69–71]. Finally, building block **23**, one important fragment in the total synthesis of amphidinolide F (**24**), was accessible in good overall yield and high diastereoselectivity (dr ≈ 95:5) in only four steps (Scheme 7).

### Oxidative cyclizations in the synthesis of annonaceous acetogenins

#### *cis*-Solamin A

**Scheme 8:**
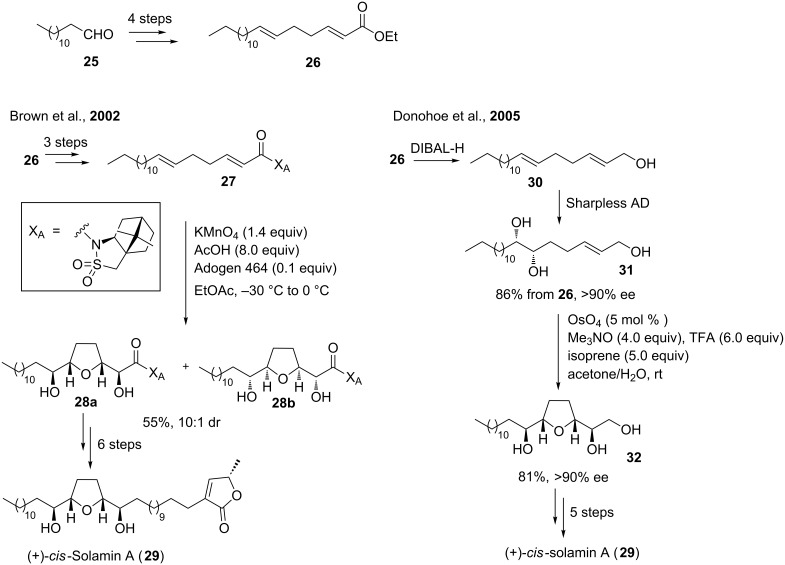
Brown`s and Donohoe`s oxidative cyclization approach to *cis*-solamin A.

*cis*-Solamin represents a typical mono-THF acetogenin, originally isolated from the roots of the tropical fruit tree *Annona muricata* in 1998 [72]. The relative stereochemistry within the THF diol core was assigned as *threo-cis-threo*, whereas the absolute configuration present in *cis*-solamin was not established at the time of isolation. Then in 2006, the groups of Figadère and Brown were able to show that natural *cis*-solamin actually occurs as a mixture of two tetra-epimeric diastereoisomers *cis*-solamin A (**29**, Scheme 8) and *cis*-solamin B [73]. It therefore has to be noted that structure **29** was referred to as “*cis*-solamin” in the literature, up to that important discovery by Figadère and Brown. Its diverse biological activities [72] together with its broadly unexplored biogenesis [73–74] motivated many synthetic groups to develop total syntheses of *cis*-solamin A (**29**) [75–83] and B [76,78,83].

In 2002, Brown and co-workers achieved concise total syntheses of *cis*-solamin A (**29**) and B using the diastereoselective, auxiliary-controlled, permanganate-promoted type A oxidative cyclization of 1,5-dienes to create the THF diol backbone and to introduce four of the five stereogenic centers present in these mono-THF acetogenins (left, Scheme 8) [76,78]. Starting from commercially available aldehyde **25**, diene **27** was obtained in few steps and subsequently cyclized. Previously established standard conditions using acetone–water delivered THF-diol **28a** in only 18% yield. Better results were achieved when the oxidative cyclization was carried out under phase-transfer conditions [84]. Thus, the corresponding THF diols were obtained in 55% yield. In addition to the desired THF diol **28a** for the total synthesis of *cis*-solamin A (**29**), small amounts of its diastereoisomer **28b** were isolated (dr 10:1, Scheme 8 left). Similar permanganate-mediated oxidative cyclizations were also successfully applied to the total syntheses of two more mono-THF acetogenins, *cis*-uvariamicin I and *cis*-reticulatacin, by the Brown group [85].

A formal synthesis of *cis*-solamin A (**29**) was published in 2005 by the Donohoe group, employing their Os(VI)-catalyzed oxidative cyclization of 5,6-dihydroxyalkenes as the key step (right, Scheme 8) [79]. After reduction of the ester **26**, a Sharpless asymmetric dihydroxylation (AD) [86–88] reaction furnished diol **31** with a high degree of both regio- and enantioselectivity. Osmium-promoted oxidative type B cyclization of **31** proceeded in high yield (81%) and with high stereoselectivity (ee >90%) to give THF diol **32**. The latter could be almost quantitatively converted to the corresponding tosylate, an intermediate in Brown`s synthesis of *cis*-solamin A (**29**) [76,78], thus completing a formal synthesis of this natural product (Scheme 8).

**Scheme 9:**
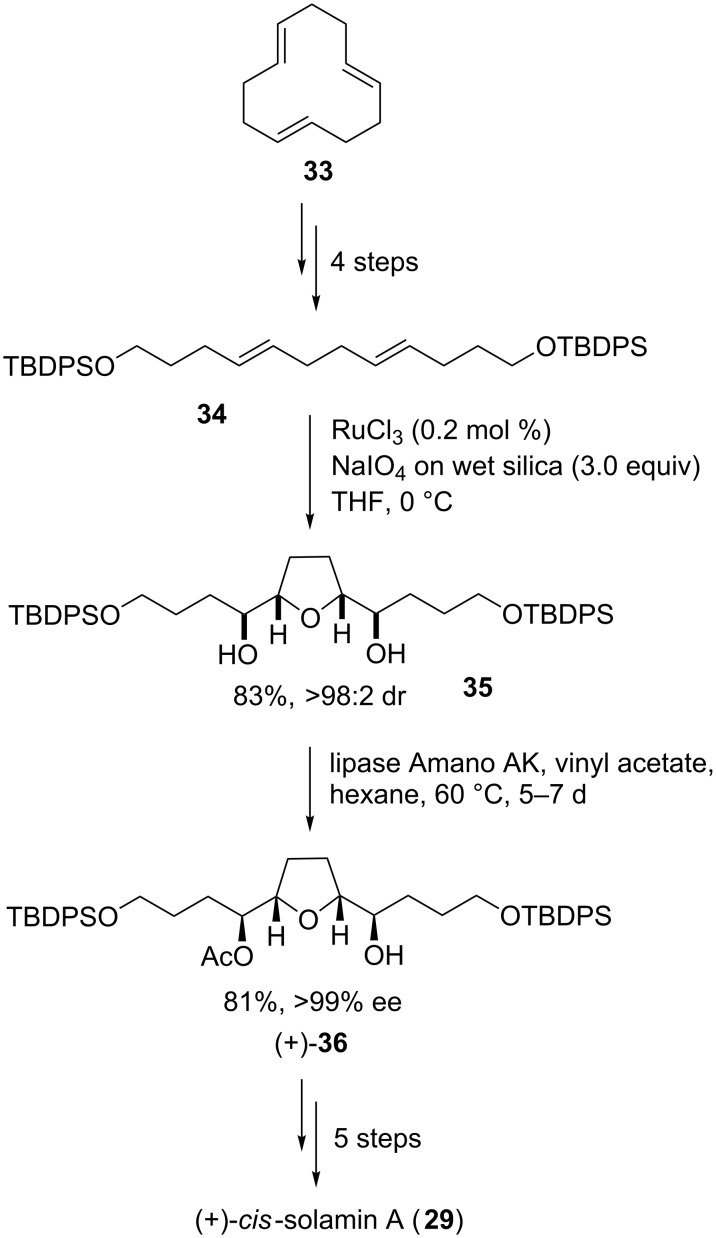
Total synthesis of *cis*-solamin A using a Ru(VIII)-catalyzed oxidative cyclization and enzymatic desymmetrization as the key steps.

In 2006, our group succeeded in synthesizing *cis*-solamin A (**29**) utilizing a ruthenium tetroxide-catalyzed type A oxidative cyclization approach (Scheme 9) [80]. Silyl-protected dienediol **34**, the oxidative cyclization precursor, was synthesized from *all*-*trans*-cyclododecatriene **33** in four steps including dihydroxylation, glycol cleavage [89], subsequent borohydride reduction and protection of the resulting diol. The Ru(VIII)-catalyzed oxidative cyclization in the presence of sodium periodate on wet silica [90] as the oxidizing agent delivered the THF diol **35** in high yield (83%). The product was formed with excellent diastereocontrol (dr >98:2). Subsequent enzymatic desymmetrization [91] using lipase Amano AK gave the enantiomerically pure acetate **36** in 81% yield (ee >99%). A further three transformations then delivered *cis*-solamin A (**29**). Crucial to the success of this approach and its high efficiency is that it takes advantage of the *meso*-geometry of the central THF diol moiety [80].

#### *cis*-Sylvaticin

**Scheme 10:**
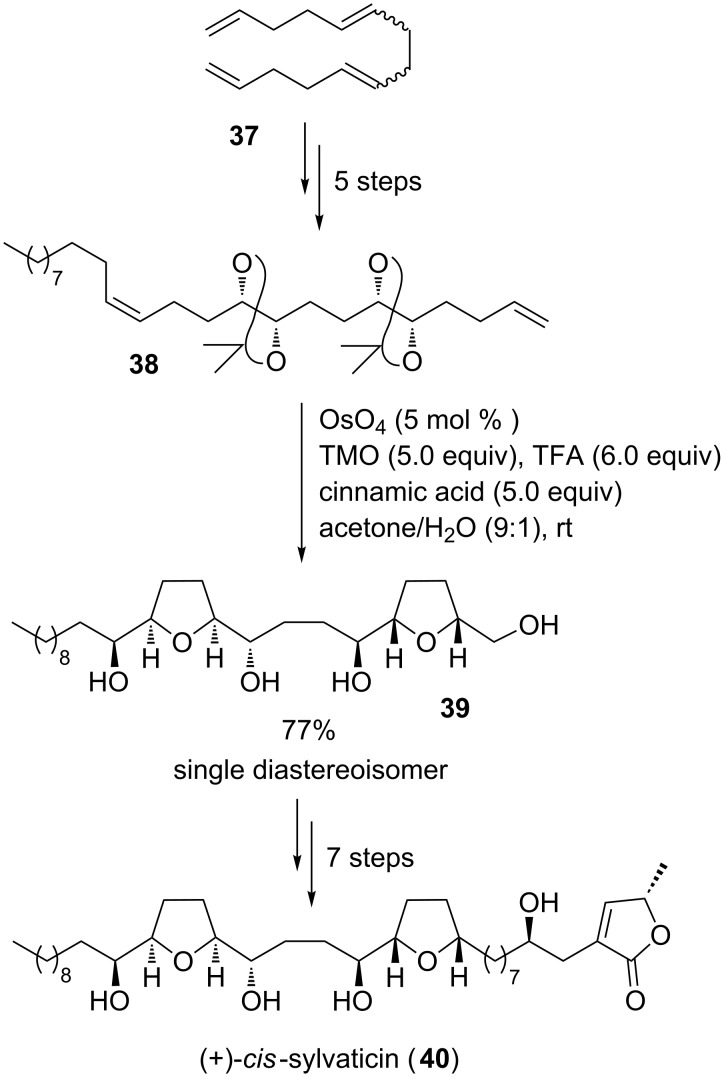
Donohoe´s double oxidative cyclization approach to *cis*-sylvaticin.

*cis*-Sylvaticin (**40**), a non-adjacent bis-THF acetogenin [92] (Scheme 10), was discovered in dried fruits of *Rollinia sylvatica* [93] and leafs of *Rollinia mucosa* [94]. It has been shown to be cytotoxic against several cancer cell lines at nanomolar concentrations [93–94]. Two different synthetic approaches to *cis*-sylvaticin (**40**) were reported, utilizing an oxidative cyclization to stereoselectively establish the *cis*-configured 2,5-disubstituted THF rings.

The first total synthesis of *cis*-sylvaticin (**40**) has been accomplished in 2006 by the Donohoe group, using an osmium-catalyzed double type B oxidative cyclization strategy (Scheme 10) [5,95]. The protected precursor **38** was synthesized from tetraene **37** in five steps involving a highly position- and stereoselective Sharpless AD reaction [86–88] (ee >98%, de >90% for the all *syn*-isomer). Subsequent osmium tetroxide-catalyzed oxidative cyclization under acidic reaction conditions resulted in bis-THF **39** which was isolated in 77% yield and as a single diastereoisomer. Thus, both THF rings of the natural product were established at the same time (Scheme 10) [5,95]. Based on this approach, in 2009, Donohoe and co-workers also reported the first total synthesis of (+)-sylvaticin [92,96], the C12-epimer of *cis*-sylvaticin (**40**) using oxidative cyclization chemistry to establish both the 2,5-*cis*- and the 2,5-*trans*-substituted THF ring of the natural product. However, it has to be noted that the *trans*-THF was not directly formed in an oxidative cyclization reaction but rather through a subsequent sequential solvolysis/hydride shift/intramolecular reduction cascade.

**Scheme 11:**
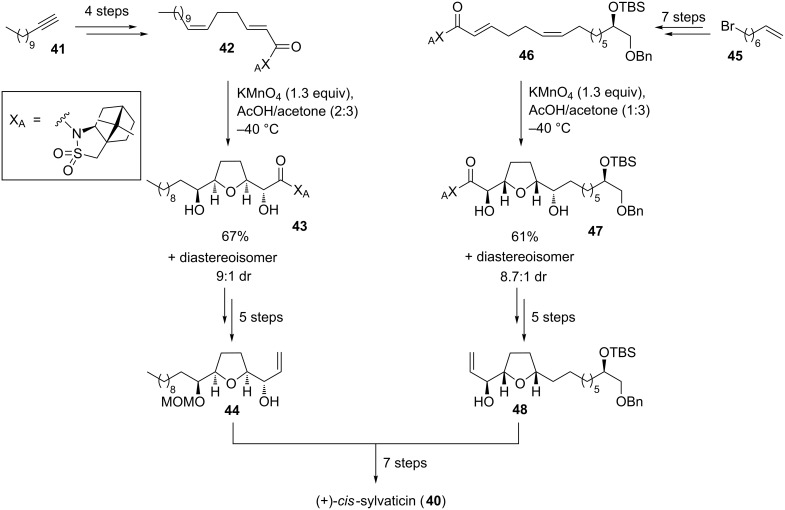
Permanganate-mediated approach to *cis*-sylvaticin by Brown and co-workers.

Another total synthesis of *cis*-sylvaticin (**40**) has been published by Brown and co-workers in 2008 [92,97]. In this case, two permanganate-promoted type A oxidative cyclization reactions were used to establish the two THF rings of this acetogenin (Scheme 11). Both THF diols **43** and **47** were isolated as pure diastereoisomers with high diastereocontrol (dr 9:1 for **43** and dr 8.7:1 for **47**, respectively) and then successfully connected in a silicon-tethered ring closing metathesis (RCM) [98] to provide the main backbone of *cis*-sylvaticin (**40**). Moreover, in 2009, Brown and co-workers reported on a short synthesis of the non-adjacent bis-THF core of *cis*-sylvaticin (**40**) making use of a permanganate-mediated bidirectional oxidative cyclization approach [99].

#### Membranacin and membrarollin

**Scheme 12:**
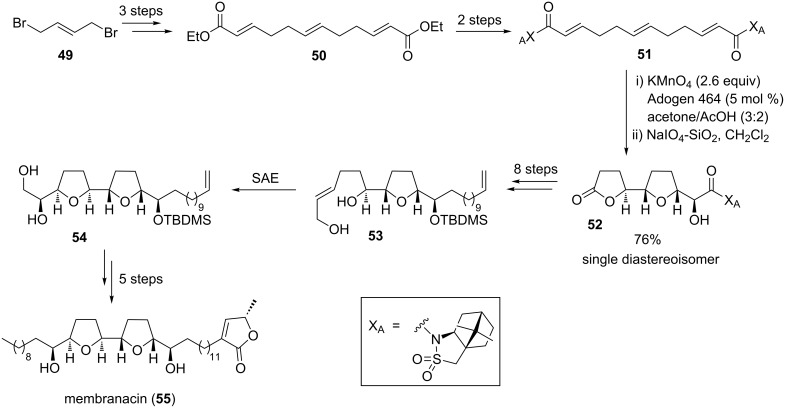
Total synthesis of membranacin using a KMnO_4_-mediated oxidative cyclization.

**Scheme 13:**
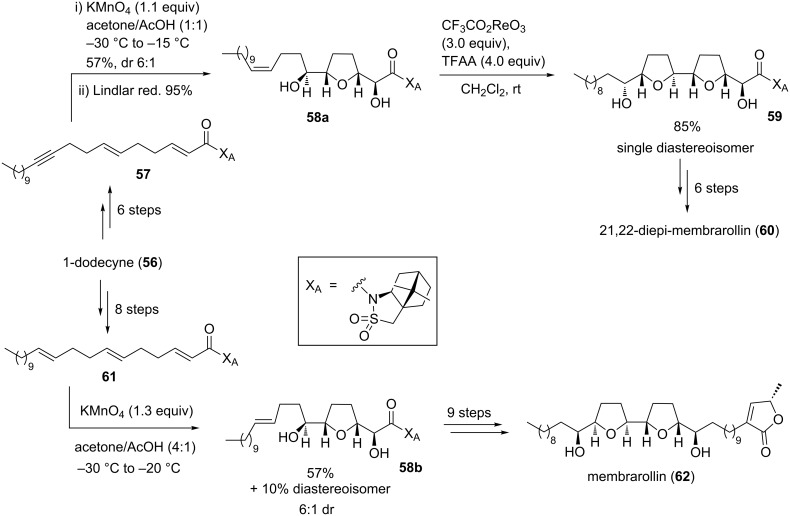
Total synthesis of membrarollin and its analogue 21,22-diepi-membrarollin.

Membranacin (**55**) and membrarollin (**62**) are typical adjacent bis-THF acetogenins having a *threo-cis-threo-cis-erythro* configured core (Scheme 12 and Scheme 13). They were isolated from the seeds of the fruit tree *Rollinia membranaceae* by the Cortes group [100–101]. Previous studies demonstrated, that particularly adjacent bis-THF acetogenins exhibit highly potent tumor growth inhibitory activity. Detailed investigations into the mode of action revealed that acetogenins inhibit cancer cell growth through the blockage of the mitochondrial NADH-ubiquinone oxidoreductase of complex I of the respiratory chain. In fact, membranacin (**55**) and membrarollin (**62**) are amongst the most potent complex I inhibitors identified to date [101]. As part of their studies towards the synthesis of adjacent bis-THF acetogenins including membranacin (**55**) and membrarollin (**62**), Brown and co-workers considered a two-stage cyclization approach to control the stereochemistry within the THF backbone ring system.

The total synthesis of membranacin (**55**), developed in 2004 by Brown and co-workers, comprised metal-oxo and metal-peroxy-mediated oxidative cyclizations as the key steps [102] (Scheme 12). Thus, permanganate oxidation of triene **51**, which can be synthesized in a few steps from (*E*)-1,4-dibromobut-2-ene (**49**), followed by treatment of the crude reaction mixture with NaIO_4_–SiO_2_ proceeded efficiently to afford the single isolated diastereoisomeric lactone **52** in 76% yield. This lactone (**52**) was converted to enediol **53** in a further few steps. The second THF ring was then established using an epoxidation–cyclization sequence. Thus, asymmetric Sharpless epoxidation (SAE) [103–104] yielded an intermediary oxirane (not shown in Scheme 12) which was intramolecularly trapped by attack of the remote hydroxy group to afford bis-THF **54**, a key intermediate *en route* to membranacin (**55**) (Scheme 12) [102].

One year later, in 2005, Brown and co-workers achieved a total synthesis of 21,22-diepi-membrarollin (**60**) [105], possessing an adjacent bis-THF motif present in various acetogenins (e.g. carolin A [106]), by applying sequential metal-oxo mediated oxidative cyclizations to introduce six of the seven stereogenic centers (Scheme 13). The required dienyne **57** was prepared from commercially available 1-dodecyne (**56**). Permanganate-promoted oxidation of dienyne **57** proceeded rapidly and selectively at low temperatures, affording the corresponding diastereomeric THF diols as a separable mixture (dr 6:1, major stereoisomer shown in Scheme 13). Semi hydrogenation of the triple bond using the Lindlar catalyst gave the bis-homoallylic alcohol **58a**, which underwent an efficient acyl perrhenate-mediated hydroxy-directed oxidative cyclization to afford a single isolated bis-THF **59** in excellent yield. A few subsequent steps were required to finish the synthesis of 21,22-diepi-membrarollin (**60**), notably avoiding the requirement for any hydroxy protecting groups.

The first total synthesis of membrarollin (**62**, Scheme 13) was finally disclosed by Brown and co-workers in 2009 [107]. Similarly starting from 1-dodecyne (**56**), triene system **61** was selectively oxidized using a permanganate-mediated oxidative cyclization affording two separable diastereoisomeric THF diols in 67% yield (only major isomer **58b** shown in Scheme 13). It is worth noting that this oxidative cyclization proceeded with high chemoselectivity leaving the remote C–C-double bond unreacted. For the formation of the adjacent THF ring different and stereodivergent strategies were studied [107]. The relative and absolute stereochemistry required to prepare natural membrarollin (**62**) was obtained using a perrhenate-mediated type B’ cyclization of THF diol **58b** (not shown in Scheme 13).

#### Rollidecin C and D

**Scheme 14:**
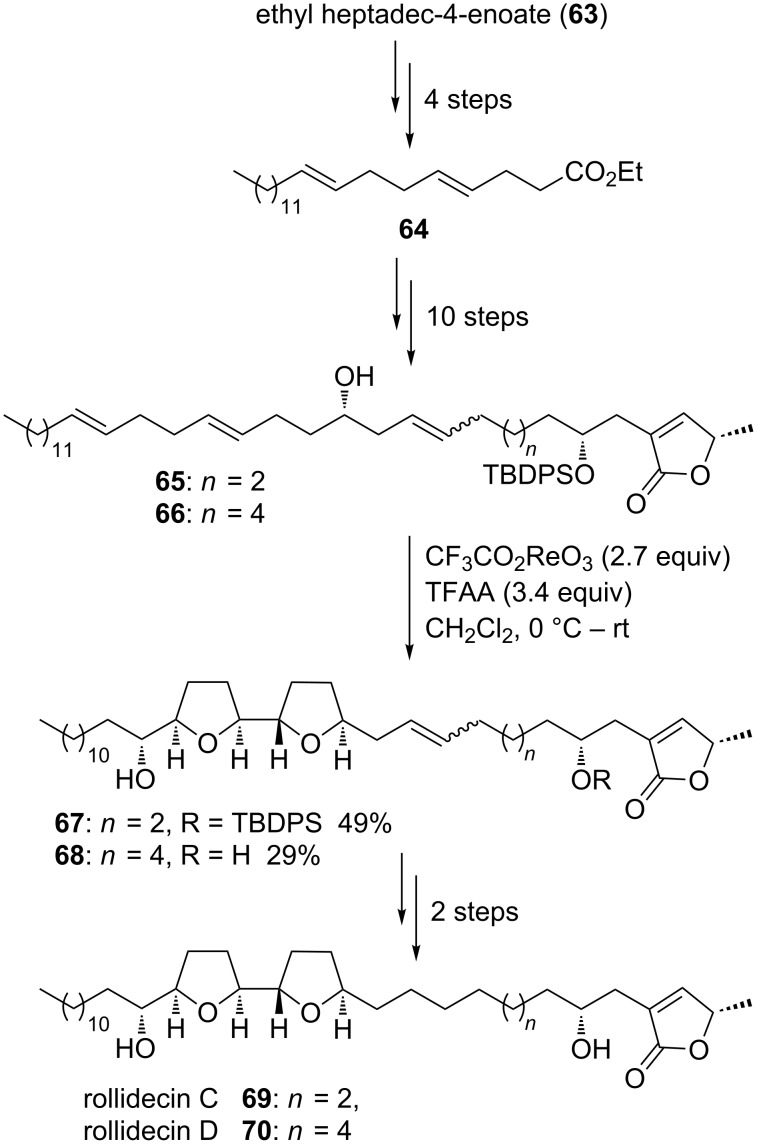
Total synthesis of rollidecin C and D using a late stage Re(VII)-catalyzed oxidative polycyclization.

Rollidecin C (**69**) and D (**70**) belong to the class of adjacent bis-THF acetogenins. In contrast to other representatives of this subgroup of acetogenins they are lacking one of the secondary alcohols usually framing the bis-THF core (Scheme 14). They were isolated from the leaves of *Rollinia mucosa* [108] and shown to exhibit cytotoxicity against six human tumor cell lines. Rollidecin C (**69**) was found to be more potent than rollidecin D (**70**) with selectivity toward the colon cell line HT-29 [108]. In 2001, the groups of Sinha and Keinan reported on a stereoselective synthesis of rollidecin C (**69**) and D (**70**) [109] using the tandem oxidative polycyclization reaction with trifluoro-acetylperrhenate, a synthetic method first reported in 1995 [110–111]. Bis-homoallylic dienols **65** and **66** were synthesized from *trans*-ethyl heptadec-4-enoate (**63**) via diene **64**. A Re(VII)-mediated type C oxidative cyclization furnished the bis-THF products **67** and **68** in 49% and 29% yield, respectively. Both THF rings were introduced with excellent diastereoselectivity in a single step transformation at the final stages of the total syntheses of rollidecin C (**69**) and D (**70**) (Scheme 14).

Similar rhenium-mediated type C oxidative cyclizations were also successfully applied in total syntheses of further acetogenins by Sinha and Keinan, e.g., asimicin [112–113], bullatacin [112–114], trilobacin [115] and even to the tris-THF acetogenins goniocin [116] and cyclogoniodenin T [116].

#### Asimilobin and gigantetrocin A

**Scheme 15:**
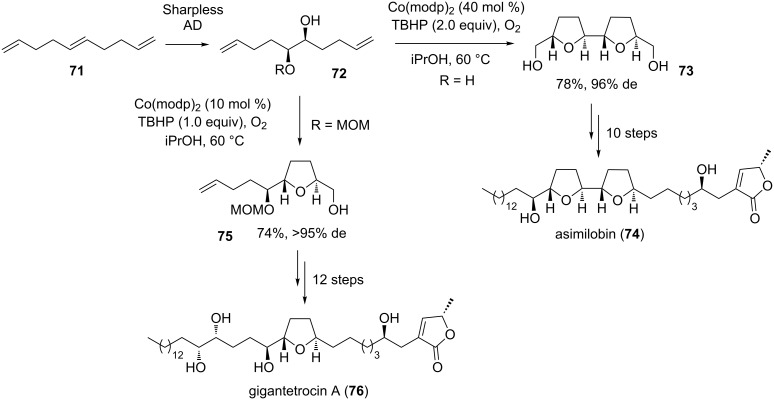
Co(II)-catalyzed oxidative cyclization in the total synthesis of asimilobin and gigantetrocin A.

Asimilobin (**74**) is a bis-THF acetogenin containing two 2,5-*trans*-configured THFs [117]. It has originally been isolated from the seeds of *Asimina triloba* [118] but has also been found in extracts of the bark of *Goniothalamus giganteus* (Annonaceae) [119] by McLaughlin and co-workers [120–121]. In 1999, Wang and Shi et al*.* disclosed the first total synthesis of (–)-asimilobin (**74**) and its diastereomer using a highly efficient and stereocontrolled synthetic strategy to construct the desired bis-THF ring building block **73** in two steps (Scheme 15) [120–121]. Thus, starting from commercially available *trans*-1,5,9-decatriene (**71**) a stereo- and positionselective Sharpless AD reaction [86–88] provided *C*_2_-symmetric diol **72** (R = H) in high selectivity (ee >94%). Subsequent Co(II)-mediated oxidative type B’ cyclization of dienediol **72** (R = H) proceeded in good yield (78%) and with high diastereoselectivity (de 96%) to give *C*_2_-symmetric bis-THF product **73** (Scheme 15). The natural product was then assembled in a further 10 steps (Scheme 15) [120–121].

Subsequently, Shi and co-workers successfully applied their synthetic strategy to the first total synthesis of gigantetrocin A (**76**) [122–123], a mono-THF acetogenin, isolated from *Goniothalamus giganteus* by McLaughlin and co-workers [124]. This time, mono-protected dienediol **72** (R = MOM) was cyclized to form *trans*-THF compound **75** in 74% yield (de >95%) using Co(modp)_2_ as a catalyst under oxygen atmosphere (Scheme 15). Finally, the synthesis of gigantetrocin A (**76**) has been achieved in seventeen steps from chiral mono-protected dienediol **72** (R = MOM) [122–123].

Further acetogenins which have been synthesized through Co(II)-mediated type B’ oxidative cyclizations include mucocin, a known mono-THF representative, by Evans et al*.* [125] and the bis-THF acetogenin bullatacin by Pagenkopf and co-workers [126]. The latter group also employed this methodology in the total synthesis of aplysiallene [127], and more recently to bovidic acid [128] and cyclocapitelline [129].

### Oxidative cyclizations in the synthesis of terpenoid natural products

#### Linalool oxide

**Scheme 16:**
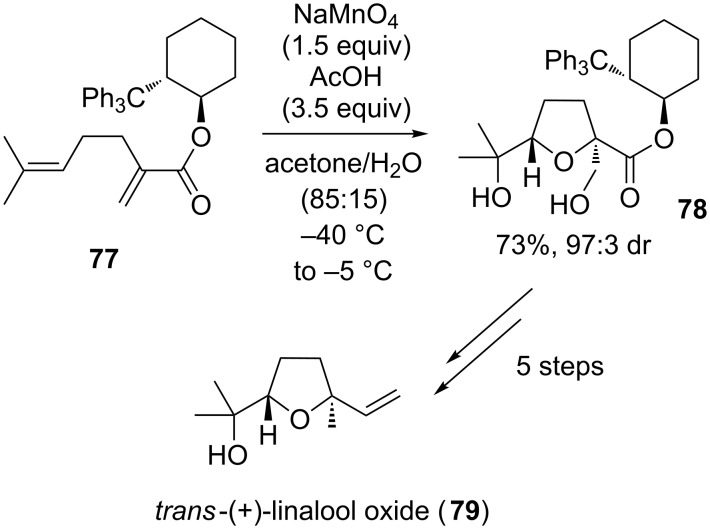
Mn(VII)-catalyzed oxidative cyclization of a 1,5-diene in the synthesis of *trans*-(+)-linalool oxide.

The monoterpenoid *trans*-(+)-linalool oxide (**79**), containing a 2,2,5-trisubstituted THF ring, can be found in food and beverages as well as essential oils and is used as powerful sweet-woody penetrating aroma component in the perfume and flavoring industry [130]. Several syntheses have been published between 1981 and 2010 using a range of different strategies (e.g. enzymatic procedures, Sn- and Pd-catalyzed methods or even anodic oxidations) [131–136]. In 2014 the Brown group proposed an auxiliary-controlled synthesis of *trans*-(+)-linalool oxide (**79**) using a permanganate-mediated type A oxidative cyclization as the key step (Scheme 16) [137]. Thus, 1,5-diene **77** was subjected to an oxidative cyclization using stoichiometric amounts of sodium permanganate to furnish *trans*-THF diol **78** in 73% yield with an excellent diastereomeric ratio of 97:3 induced by a cyclohexanol derived chiral auxiliary. This key intermediate was subsequently converted to natural *trans*-(+)-linalool oxide (**79**) in a further few steps.

#### Teurilene

**Scheme 17:**
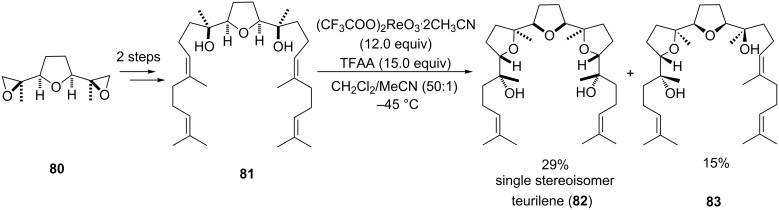
Re(VII)-catalyzed oxidative cyclization in the total synthesis of teurilene.

Teurilene (**82**) is a squalene-derived cytotoxic polyether which was originally extracted from the red algae *Laurencia obtusa* by Suzuki et al. [138–139]. Though it is *C*_S_-symmetric, it is structurally closely related to pentacyclic *C*_2_-symmetric glabrescol [140], another triterpene natural product found in Jamaican endemic plant *Spathelia glabrescens* (Rutaceae) [141]. In 1999, Morimoto and co-workers reported on a stereoselective synthesis of the *meso*-tris-THF natural product teurilene (**82**) [142–143] (several previous total syntheses existed [144–151]) using an elegant two-directional approach (Scheme 17).

Thus, starting from a central THF diol **81** with a fully established carbon framework, which was derived from *C*_S_-symmetric bis-epoxide precursor **80**, a double oxidative cyclization using Re(VII)-catalysis furnished the natural product in 29% yield (Scheme 17). This (supposedly) type B’ ring forming reaction occurred with high stereoselectivity for the *trans*-isomer and in addition a minor amount of the mono-cyclization product **83** was obtained.

#### Eurylene

**Scheme 18:**
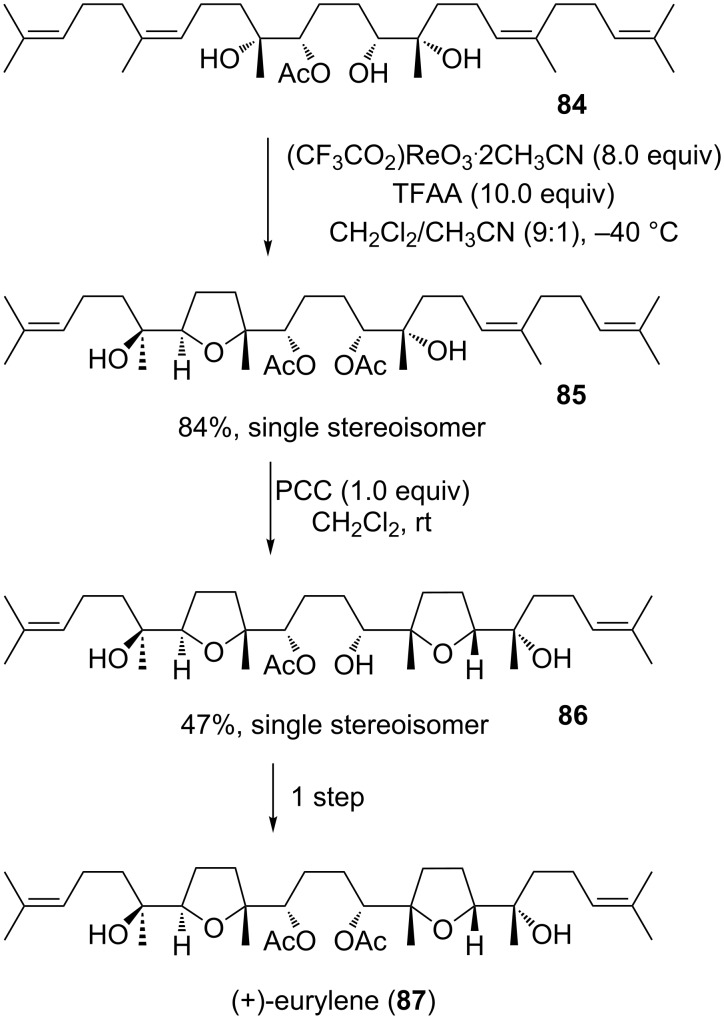
Total synthesis of (+)-eurylene via Re(VII)- and Cr(VI)-mediated oxidative cyclizations.

Eurylene (**87**) represents yet another oxasqualenoid triterpene, sharing some structural similarity with teurilene and glabrescol, but other than the latter two, eurylene (**87**) is neither *C*_S_- nor *C*_2_-symmetric (Scheme 18). It has been isolated from the wood of *Eurycoma longifolia* by Itokawa et al. in 1991 [152] and was shown to exhibit cytotoxic properties against lymphocytic leukemia. The first total synthesis by Ujihara et al. [153] followed five years after its original discovery.

In 2000, the Morimoto group developed a total synthesis using two type B’ cyclization steps (Re(VII) and Cr(VI) catalysis) to form the THF-heterocycles of the natural product (Scheme 18) [154]. The linear precursor **84** was cyclized diastereoselectively to the mono-THF intermediate **85** with an oxorhenium(VII) complex and was subsequently subjected to the second oxidative cyclization using stoichiometric amounts of pyridinium chlorochromate (PCC) to give the bis-THF compound **86**, which was easily converted to enantiomerically pure (+)-eurylene (**87**) (Scheme 18).

**Scheme 19:**
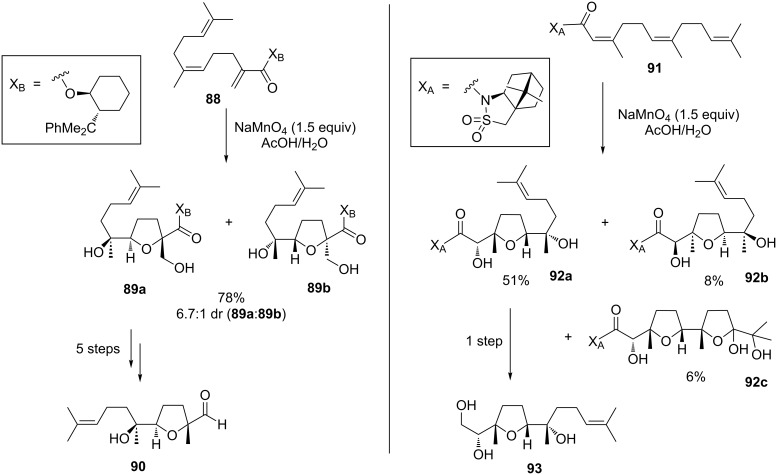
Synthesis of *cis*- and *trans*-THF Rings of eurylene via Mn(VII)-mediated oxidative cyclizations.

The Brown group published an enantioselective synthesis of the *cis*- and *trans*-THF fragments of eurylene (**87**) in 2010 [155] using an auxiliary controlled Mn(VII)-promoted oxidative cyclization to form THFs **90** and **93** (Scheme 19). Both THF-derivatives had previously been prepared in a different approach and used as intermediates in a total synthesis of eurylene (**87**) by Kodama and co-workers [156]. Brown’s permanganate mediated oxidative cyclization of precursor **88** gave a yield of 78% and a diastereomeric ratio of 6.7:1 in favor for the desired product **89a**. Though this reaction is *cis*-selective, cunningly, deoxygenation ultimately leads to the *trans*-THF fragment **90**. The other THF subunit **93** of the natural product **87** was prepared via an oxidative mono-cyclization of triene **91**. Thus, the desired *cis*-product **92a** was obtained in 51% yield together with 8% of its diastereoisomer **92b** and a minor amount of the double cyclized product **92c**. Both synthesized THF fragments were consistent with those reported by Kodama [156] (Scheme 19).

#### Venustatriol

**Scheme 20:**
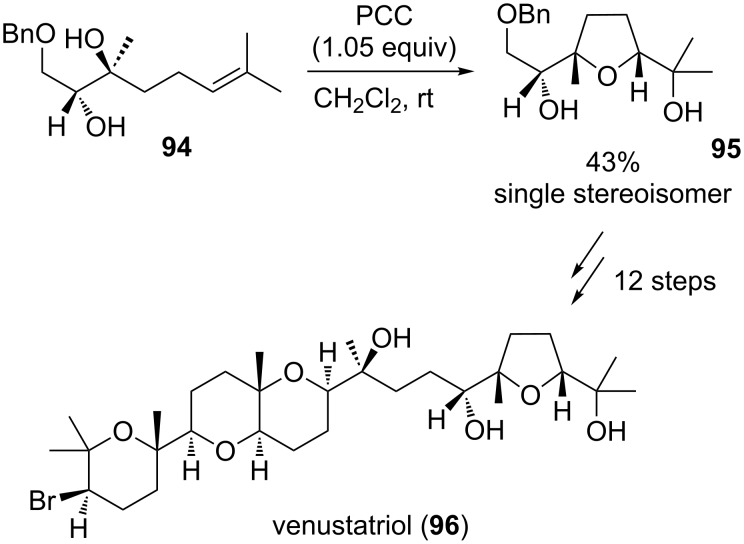
Cr(VI)-catalyzed oxidative cyclization in the total synthesis of venustatriol by Corey et al.

The tetracyclic oxasqualenoid venustatriol (**96**) was isolated in 1986 by Sakemi et al. from the red algae *Laurentia venustra* and exhibited antiviral activity against vesicular stomatitis virus (VSV) and herpes simplex virus type 1 (HSV-1) [157]. Hashimoto et al. reported a total synthesis of the natural product in 1988 [158–159] employing a vanadium-catalyzed epoxidation as a key step in the stereoselective formation of the THF ring, whilst the Corey group achieved a total synthesis using a PCC-mediated oxidative type B cyclization in the same year (Scheme 20) [160].

Diol **94**, derived from geraniol, was diastereoselectively converted into the THF derivative **95** in a yield of 43% using an oxochromium(VI) complex. Venustatriol (**96**) could then be obtained by C–C-coupling with the corresponding THP fragment in an enantioselective fashion.

#### Glaciapyrrol A

**Scheme 21:**
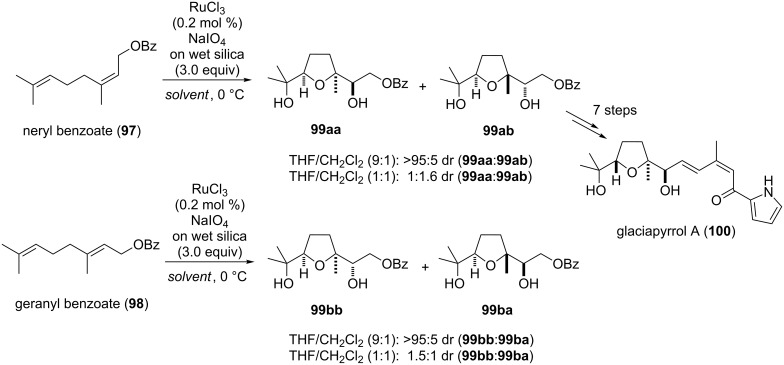
Ru(VIII)-catalyzed oxidative cyclization of a 1,5-diene in the synthesis and evaluation of its stereochemistry of glaciapyrrol A.

Glaciapyrrol A (**100**), B and C form a family of pyrrolo sesquiterpenoids which have been isolated in 2005 from a marine *Streptomyces sp.* (NPS008187) by Macherla et al. [161]. The only established total synthesis has been developed by the Dickschat group in 2011 [162] using a type A Ru(VIII)-catalyzed oxidative cyclization as the key step (Scheme 21).

Both neryl benzoate (**97**) as well as geranyl benzoate (**98**) have been converted into the corresponding THF diols **99** using an established oxidative cyclization protocol [15,163–164]. The diastereoselectivity of the reaction varied depending on the solvent composition used [15]. Therefore, reaction of **97** using THF/dichloromethane (9:1) as the solvent mixture resulted in a selective formation of *cis*-THF **99aa**, whereas a 1:1 mixture of the same solvents gave a diastereomeric ratio of 1:1.6 in favor for the *trans*-isomer **99ab**. Oxidative cyclization of *trans*-configured starting material **98** proceeded with similar efficiency. In this case a 9:1 solvent ratio gave **99bb** selectively and a 1:1 solvent mixture resulted in a diastereomeric ratio of 1.5:1, favoring **99bb** (Scheme 21). Glaciapyrrol A (**100**) was finally obtained from **99ab** by deprotection of the benzoyl group and an olefination to connect the pyrrole subunit of the natural product.

#### Leucosceptroids A–D

**Scheme 22:**
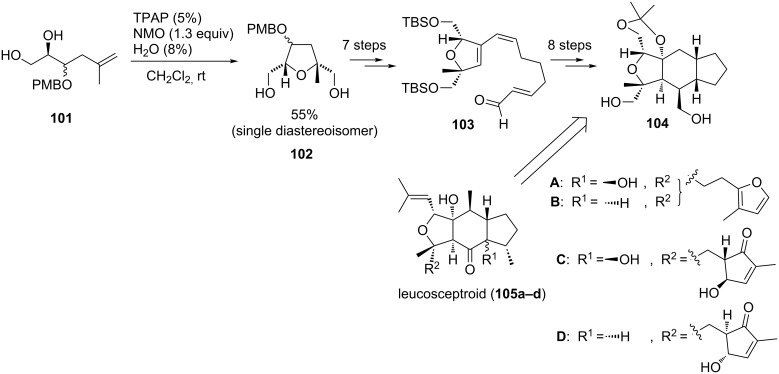
Ru(VII)-catalyzed oxidative cyclization of a 5,6-dihydroxy alkene in the synthesis of the core structure of the leucosceptroids A-D.

Leucosceptroids A (**105a**) and B (**105b**) have been isolated in 2010 by Luo et al. from the Chinese shrub *Leucosceptrum canum* [165]. One year later the same group was able to isolate two additional leucosceptroids C (**105c**) and D (**105d**) from the leaves of the same plant [166]. This family of sesterterpenoids is believed to be beneficial to the plant as part of a defense mechanism against herbivores. The first total synthesis of leucosceptroid B (**105b**) was established in 2013 by Huang et al. [167] and two other total syntheses of leucosceptroids A (**105a**) and B (**105b**) followed two years later [168–169]. The common tricyclic core structure of the natural products had already been synthesized in 2011 by the Horne group [170], using a TPAP-catalyzed type B oxidative cyclization to form the densely substituted THF diol motif following a protocol of our group [25] (Scheme 22).

The 5,6-dihydroxyalkene **101** was obtained from D-mannitol diacetonide via oxidation and C-C-bond formation. The oxidative cyclization catalyzed by Ru(VII) yielded THF diol **102** in 55% yield as a single diastereoisomer, without considering the configuration of the protected alcohol, as this position was subsequently oxidized to enable a Sonogashira cross-coupling to access **103**. The tricyclic core structure **104** could be obtained via an intramolecular Diels–Alder reaction, epoxidation and protection (Scheme 22) [170].

## Conclusion

The direct oxidative cyclization of 1,5-dienes is known for more than 90 years, since the early finding by Kötz and Steche in 1924. While mechanistic and stereochemical aspects were in the center of research for many years, during the last two or three decades this unusual reaction has been advanced to a powerful and reliable strategy to establish 2,5-disubstituted *cis*-THF diols from very simple (often achiral) diene substrates. The reaction proceeds with a substantial increase in structural and stereochemical complexity from the starting material to the product. A similar development can be stated for the mechanistically closely related oxidative cyclizations of 5,6-dihydroxyalkenes and 5-hydroxyalkenes. All these processes are stereochemically predictable and the double bond geometry dictates the relative vicinal hydroxy ether stereochemistry at both sides adjacent to the THF-oxygen. 2,5-Disubstituted *trans*-THFs are still significantly harder to prepare using these strategies or require specific procedures. Similarly, the control of the absolute stereochemistry remains a challenge to be solved in future investigations. To date there is only a single report on an enantioselective oxidative cyclization of a 1,5-diene using permanganate together with a chiral counter ion. Other strategies use either a chiral auxiliary, a subsequent desymmetrization or start from a chiral 5,6-dihydroxy alkene or 5-hydroxy alkene substrate and proceed in a diastereoselective fashion to yield optically pure products. Though the latter procedures are quite powerful, the development of a catalytic asymmetric oxidative diene cyclization appears still a worthwhile task to be solved. Moreover, one can expect that future applications in target-oriented and natural product synthesis will also apply the same reaction methodology for the construction of THP or even oxepan compounds.

Overlooking these future directions, the direct oxidative cyclization of 1,5-dienes and mechanistically related oxidative THF forming reactions seem now to be firmly established methods for the application in complex total synthesis and are expected to deliver further exciting examples. More than 25 successful examples from the past three decades from different classes of natural products (including carbohydrates, polyketides, amino acids, fatty acids as well as acetogenins and terpenoids) are summarized in this review article.
